# Predicting Psychopathological Onset: Early Signs of Neuropsychiatric Diseases

**DOI:** 10.3390/jpm12050778

**Published:** 2022-05-11

**Authors:** Marco Costanzi

**Affiliations:** Department of Human Sciences, LUMSA University, 00193 Rome, Italy; m.costanzi@lumsa.it

Millions of people worldwide are affected by neuropsychiatric disorders, such as anxiety, major depression, bipolar disorder, schizophrenia, obsessive–compulsive disorder, autism spectrum disorders, eating disorders, addiction, and dementia. The identification of the early signs of these pathologies is an important goal to be reached in order to improve treatment effectiveness and to prevent poor outcomes.

The aim of this Special Issue is to collect valuable contributions from scientists worldwide working on the role that biological, behavioral, and cognitive markers can have in predicting the onset of neuropsychiatric disorders. We were able to collect 13 original articles and 2 reviews on this topic. The results published in this Special Issue could provide significant support in pre-clinical phases for the identification of vulnerability factors, to better understand the course of the illness, and to predict its outcome, as well as aiding clinicians in the therapeutic decision-making process.

The observation that patients suffering from a rare metabolic syndrome, trimethylaminuria, also show excessive fear, anxiety, social phobia, a sense of marginalization, suicidal ideation, a sense of persecution, and mood alterations seems to provide an interesting biological scenario linking the mind–body system to mental illness. Notably, gut microbiota alterations, which are responsible for the onset of metabolic syndrome, result in dysfunctions of neurotransmitter release and vagus nerve activation, which might determine the widest spectrum of the psychiatric disorders shown by the affected patients. Therefore, the microbiota–gut–brain axis may become a potential new target for improving the treatment of neuropsychiatric disorders [1]. Cattaneo and collaborators, by reviewing the literature in the field, suggest that the heart–brain relation is important in understanding the etiopathogenetic mechanisms of several psychopathologies and in pursuing mental health. In their work, the authors suggest an interesting relationship between the stress level of an organism and persistent alterations in the neurovegetative system, including the vasovagal system, which, in turn, results in a low heart rate variability. Such a low heart rate variability correlates with emotional dysregulation and frontal lobe dysfunctions, which are considered hallmarks of psychopathological dimensions [2].

Prefrontal cortex circuits are mainly involved in executive functions, such as the inhibitory control mechanisms that control active forgetting processes. Active forgetting plays a pivotal role in suppressing stressful intrusive memories. The suppression of these unwanted memories appears to be critical in preserving mental health, whereas deficits in the inhibitory control of these memories correlate with several psychopathological disorders, such as depression, schizophrenia, post-traumatic stress disorder, and obsessive–compulsive disorder [3].

The results discussed in the abovementioned papers provide an interesting scenario in which the interplay between the biological, psychophysiological, cognitive, and affective domains should be carefully taken into account when considering the possibility of predicting the onset of neuropsychiatric disorders.

The association between genetic variants and several neuropsychiatric disorders has been extensively demonstrated. However, several factors (e.g., the lack of reproducibility of the genetic association data published to date, the weakness of statistical associations, the heterogeneity of the phenotype, and the massive influence of the environment on human behavior) have to be adequately considered when the role of single polymorphic variants are related to the onset of specific neuropsychiatric disorders. By selecting 24 polymorphisms in genes related to human behavior previously associated with criminal behavior, Zampatti and collaborators found that these genetic variants are not clearly associated with antisocial behavior, suggesting that environmental factors could better explain the onset of violent and criminal behaviors [4].

Therefore, it is worth considering the simultaneous presence of several factors, as well as the relationship between them, when predicting the development of psychopathological trajectories.

Emotional dysregulation (i.e., the inability to monitor and evaluate emotional experiences, as well as the inability to modulate emotional reactions to meet situational demands) and temperamental features (i.e., biological and constitutional characteristics of behavioral tendencies) appear as independent factors in predicting suicidal ideation in young adults with bipolar and depressive disorders [5]. A specific form of emotional dysregulation (namely, the alexithymia), together with body image concerns, positively correlate with exercise addiction. In this relationship, self-esteem emerges as a moderating factor, playing an important role as a protective factor [6]. Another form of addiction that particularly affects adolescents is pathological gambling. Terrone and collaborators found that a chain of multiple risk factors can predict gambling onset. Such a chain begins with an insecure attachment, which negatively influences the developmental perspective and affects the theory of mind towards one’s best friend [7]. Although restricted to specific neuropsychiatric disorders, these findings on addiction seem to provide insight into the need to consider possible relationships among several risk factors. This approach may have important clinical implications by orienting preventive activities (e.g., the formation of a positive peer relationship and performing regular exercise), as well as addressing tailored treatments for addicted individuals [6,7].

As concerns the role played by alterations in the cognitive domain in neuropsychiatric disorders, Bechi Gabrielli and collaborators revealed how deficits in executive functions may be considered a potential hallmark for bipolar disorder [8]. Euthymic bipolar patients, who are in the remission phase, show deficits in the trade-off between attentional boost and attentional competition (i.e., a lack of the attentional boost effect), suggesting that temporal selective attention processes are defective in these patients [8]. Kutz and collaborators extend the involvement of the cognitive domain in degenerative disorders, pointing out the role of motor functions. By examining the association between finger tapping and cognitive function in patients affected, or supposed to be affected, by mild cognitive impairments, they suggest that results on the diadochokinetic nature of finger tapping have to be carefully taken into account when simple finger movements are considered a hallmark of age-related neurodegeneration. The assessment of the degeneration of the relevant motor systems (e.g., the cerebellum) must be considered to establish tapping as a good classifier for predicting the onset of neurodegenerative disorders [9].

The need to consider several risk factors acting on different domains has prompted researchers to develop new tools to effectively predict the onset of neuropsychiatric disorders.

Byeon developed a nomogram that could help medical professionals in the primary care setting identify people at high risk of depression. The results of his cross-sectional study, in which elderly people underwent a comprehensive evaluation that included a health survey, blood pressure measurements, physical measurements, blood tests, and a standardized depression screening test, point out the importance of continuously monitoring complex risk factors (such as household income, skipping breakfast, moderate-intensity physical activity, subjective stress, and subjective health status) to prevent depression in older adults [10]. Conti and collaborators considered the importance of introducing neuroanatomical criteria in improving the effectiveness of early differential diagnosis and in tailoring specific early intervention in neuropsychiatric disorders that share common clinical signs. By investigating the brain morphology of children with autism spectrum disorder or childhood apraxia of speech, as well as children with typical development, they successfully applied a machine learning method able to reach an optimal predictive power to differentiate between the two pathological conditions and from typical development [11]. Gori and collaborators developed a new measurement method for the assessment of mentalizing: the Multidimensional Mentalizing Questionnaire (MMQ). In their research, the authors underlined the centrality of the mentalizing construct in different forms of neuropsychiatric disorders and proposed mentalizing as a broad and multifaceted concept that encompasses and combines multiple constructs involved in treating others and ourselves as social agents. In this framework, the MMQ can be usefully adopted in both research and clinical practice, being a valuable self-reporting tool for repeated measurements of a patient’s status over the course of therapy, favoring tailored interventions and supporting clinical research [12]. Mangialavori and collaborators, by investigating perinatal affective disorder, highlighted the importance of screening fathers in perinatal health services, which are still predominantly mother-centered, and pointed out the importance of appropriate gender-sensitive screening for detecting fathers’ affective symptoms, given the impact of men’s psychological distress on the well-being of the whole family [13].

Finally, the negative impact that the COVID-19 pandemic and the associated restrictive measures have had on mental health [14] urgently requires the development of efficient psychological interventions to prevent and tackle mental disorders in addition to adequate socio-sanitary policies aimed at limiting the pandemic [15].
